# Supplementary material for: HIVprotI: an integrated web based platform for prediction and design of HIV proteins inhibitors
Source: J Cheminform. 2018 Mar 9;10:12. doi: 10.1186/s13321-018-0266-y (PMC5845081; doi:10.1186/s13321-018-0266-y)
Supplement: Supplementary file 2 — Additional file 2. Source code of HIVProtI web server. [file 13321_2018_266_MOESM2_ESM.zip › HIVprotI_Source-code/hivprotI/weka-3-6-6/doc/weka/classifiers/trees/adtree/package-summary.html]

weka.classifiers.trees.adtree


---


|  |  |  |
| --- | --- | --- |
| **Overview** \| **Package** \| Class \| **Tree** \| **Deprecated** \| **Index** \| **Help** \| **Weka's home** | |  |
| **PREV PACKAGE**   **NEXT PACKAGE** | **FRAMES**    **NO FRAMES**     **All Classes** |  |


---

## Package weka.classifiers.trees.adtree

| **Class Summary** | |
| --- | --- |
| **PredictionNode** | Class representing a prediction node in an alternating tree. |
| **ReferenceInstances** | Simple class that extends the Instances class making it possible to create subsets of instances that reference their source set. |
| **Splitter** | Abstract class representing a splitter node in an alternating tree. |
| **TwoWayNominalSplit** | Class representing a two-way split on a nominal attribute, of the form: either 'is some\_value' or 'is not some\_value'. |
| **TwoWayNumericSplit** | Class representing a two-way split on a numeric attribute, of the form: either 'is < some\_value' or 'is >= some\_value'. |

---


|  |  |  |
| --- | --- | --- |
| **Overview** \| **Package** \| Class \| **Tree** \| **Deprecated** \| **Index** \| **Help** \| **Weka's home** | |  |
| **PREV PACKAGE**   **NEXT PACKAGE** | **FRAMES**    **NO FRAMES**     **All Classes** |  |


---
